# Study of the Relevance of the Quality of Care, Operating Efficiency and Inefficient Quality Competition of Senior Care Facilities

**DOI:** 10.3390/ijerph14091047

**Published:** 2017-09-11

**Authors:** Jwu-Rong Lin, Ching-Yu Chen, Tso-Kwei Peng

**Affiliations:** 1 Department of International Business, Tunghai University, Taichung 40704, Taiwan; jrlin@thu.edu.tw; 2 Department of Business Administration, Asia University, Taichung 41354, Taiwan; tkpeng@asia.edu.tw

**Keywords:** EBM metafrontier DEA, quality of care, operating efficiency, inefficient quality competition hypothesis

## Abstract

The purpose of this research is to examine the relation between operating efficiency and the quality of care of senior care facilities. We designed a data envelopment analysis, combining epsilon-based measure and metafrontier efficiency analyses to estimate the operating efficiency for senior care facilities, followed by an iterative seemingly unrelated regression to evaluate the relation between the quality of care and operating efficiency. In the empirical studies, Taiwan census data was utilized and findings include the following: Despite the greater operating scale of the general type of senior care facilities, their average metafrontier technical efficiency is inferior to that of nursing homes. We adopted senior care facility accreditation results from Taiwan as a variable to represent the quality of care and examined the relation of accreditation results and operating efficiency. We found that the quality of care of general senior care facilities is negatively related to operating efficiency; however, for nursing homes, the relationship is not significant. Our findings show that facilities invest more in input resources to obtain better ratings in the accreditation report. Operating efficiency, however, does not improve. Quality competition in the industry in Taiwan is inefficient, especially for general senior care facilities.

## 1. Introduction

### 1.1. Motivations

The number of people in the world over the age of 60 is expected to double by 2050. Creating long-term sustainable and age-friendly health systems requires immediate action from practitioners, researchers, and all levels of government, according to a 2015 report released by the World Health Organization [1]. Longer lives do not mean healthier lives. However, more and more of the elderly and healthy population are still active, independent, and wish to make contributions. High quality and efficient senior care institutions, providing both social and care functions, thus become important communities/facilities to ensure elderly people live in an active and secure environment.

In Taiwan, based on the report “Population Projections for the R.O.C. (Taiwan): 2016–2060”, Taiwan is projected to become an aged society in which 14% of population is 65 years and older in 2018 and a super-aged society in which 20% of population is elderly in 2026. In 2060, Taiwan will be one of the top three countries that have the highest percentage of elderly in the population in the world [2]. To ensure quality of care for seniors, the government passed the Senior Citizens Welfare Act, which regulates the scale, area, facilities, staff and scope of senior care facilities, in 2009. Article 37 of the act requires senior care facilities to be supervised, inspected, evaluated and rewarded by the central, municipal and city governments periodically in order to ensure quality and protect the rights of senior citizens and their families. 20% of the accreditation score goes to administrative management including 23 items, 40% is daily care and professional services including 38 items, 25% is facilities and environmental safety including 28 items, 13% is equity including 12 items, and 2% goes to innovation including two items. Facilities rated “A” or “B” are awarded with financial incentives and have priority for receiving government subsidies. However, there is little evidence to support the effectiveness of the accreditation system. In the literature, industries are found to invest a great deal of resources and reduce the amount of accommodation or number of inpatients in order to gain better accreditation scores. This phenomenon is called “inefficient quality hypothesis” or “overinvestment in quality” [3], which is a major issue that we discuss and examine in this study.

In this study, we construct mathematical models and conduct empirical studies to examine existing senior care facilities in Taiwan in terms of quality of care and operating efficiency. In our census data, senior care facilities in Taiwan are categorized into general (senior) care facilities and nursing homes depending on the facility’s capacity to take care of different types of self-care residents. Nursing homes only accommodate residents who can manage their basic daily life while general care facilities also accommodate elderly people without self-care ability. Considering possible differences in the nature of operations, we therefore propose to (1) evaluate and compare operating efficiency, including three technical efficiencies, for these two types of facilities by data envelopment analysis and metafrontier analysis; (2) apply a regression model to examine the relationship between operating efficiency and quality of care; and (3) investigate the influence of the quality rating in the industry.

### 1.2. Literature Review

This section details literature reviews related to our mathematical methods and hypotheses including inefficient quality competition, data envelopment analyses (DEA), and metafrontier efficiency analysis (MTE).

#### 1.2.1. Inefficient Quality Competition

Quality competition originated from the military concept of an “arms race”, which is a competition between two nations/countries each attempting to have the best equipped armed forces. In the industry, inefficient quality competition, often a result of information asymmetry, describes two firms competing to have the best quality, without any actual improvements in efficiency or productivity [3]. In the literature, quality competition has been studied in many industries. Foote [4] and James [5] discussed technical investment competition in the hospitals. White [6] investigated the arms race in the auto industry. Gritsko et al. [7] discussed the CEO arms race. Devers et al. [8] found that since year 2000, hospitals in the U.S. began non-price competition (i.e., service, quality, and equipment) which is a new type of arms race other than the price competition they had engaged in prior to 2000. The authors concluded that the medical arms race increased hospital costs and total health expenditures. Berenson et al. [9] discovered another type of medical arms race between specialty services provided by physician-owned specialty hospitals and traditional hospitals. Carey and Stefos [10] adopted patient safety indicators (PSIs) to represent health care quality. The authors incorporated PSIs into hospital cost functions and concluded that health care quality and hospital operating costs are positively related. Blank and Eggink [11] studied the relationship between hospital productivity and government policy in the Netherlands during 1972–2010 and discovered that hospital productivity was related to government regulation. Trybou et al. [12] found that there was a trade-off between administrative obligations and professional obligations (clinical excellence and physician autonomy) and the ambiguous interaction of these two obligations influenced the financial performance of hospitals in Belgium. In Taiwan, Tsai and Li [13] examined the situation of a medical arms race and concluded that medical expenditure, for example, on hi-tech medical equipment, is positively related to the degree of market competition.

#### 1.2.2. Data Envelopment Analyses (DEA)

Data envelopment analysis (DEA) is a non-parametric programming method to evaluate the relative efficiencies of organizations with multiple outputs and inputs. DEA can simultaneously estimate overall efficiency, technical efficiency, and economies of scale, distinguish inefficient units in the database, and provide target improvement values. DEA models have been applied to many industries, including the health care sector. Sexton et al. [14] evaluated the operating efficiency of nursing homes and discussed the impact of increased rate of service on operating efficiency. The authors found that the operating efficiency of profit organizations were higher than that of non-profits; however, non-profit organizations provided better quality of health care. Nyman et al. [15] constructed an output-oriented DEA model to study the relations among case-mix, resident accommodation rate and technical efficiency in nursing homes and discovered that the resident accommodation rate was the main driving force of technical efficiency. Fizel & Nunnikhoven [16] grouped nursing homes according to the intensity of care received by residents and investigated relative efficiency among groups. They also concluded that for-profit nursing homes were more efficient than non-profit ones. The same conclusions can also be found in Ozcan et al. [17]. Rosko et al. [18] examined 461 nursing homes in Pennsylvania and found that environmental factors such as ownership, occupancy rate, employee salary and payment source had more impact on operating efficiencies than service quality characteristics. Chen [19] assessed 55 chartered nursing homes in Taiwan using DEA analysis and studied the impact of ownership, scale, and length of operation. The results showed that 41% of facilities in the data were relatively efficient and that scale had the most impact on efficiency. Yang [20] used the total number of elderly and the number of available beds in nursing homes in Taiwan as variables in the DEA analysis to study environmental impact on efficiency. The author showed that efficiency rankings are unaffected by the environmental input variable but would differ if the environmental output variable is considered.

#### 1.2.3. Metafrontier Efficiency Analysis (MTE)

Metafrontier efficiency analysis has been a popular approach for comparing the efficiency of one subject in a group to another subject in a different group in the literature. Metafrontier DEA and metafrontier stochastic frontier analysis (SFA) are often used to examine whether the inefficiency is due to group-specific characteristics. We refer to Chang, Huang & Wang; Assaf et al.; Battese and Rao; Battese et al.; Rao; Lin et al.; O’Donnell et al.; Huang, Chang, and Chiu; and Yen & Chang [21,22,23,24,25,26,27,28,29]. In particular, O’Donnell et al. [27] used both metafrontier DEA and metafrontier SFA to assess firm level efficiency via mathematical programming techniques and conducted empirical studies of cross-country agricultural sector data from 97 countries. Differing from the programming techniques used in O’Donnell et al. [27], Huang et al. [30] proposed a two-step stochastic frontier approach to estimate metafrontier production function, which enabled statistical properties and inferences to be drawn.

## 2. Materials and Methods

This section is divided into four subsections. We first formulate technical efficiency and construct null hypotheses, followed by three mathematical models: data envelopment analysis (DEA), metafrontier efficiency analysis (MTE), and regression models.

### 2.1. Technical Efficiency and Null Hypotheses

**Figure 1:**
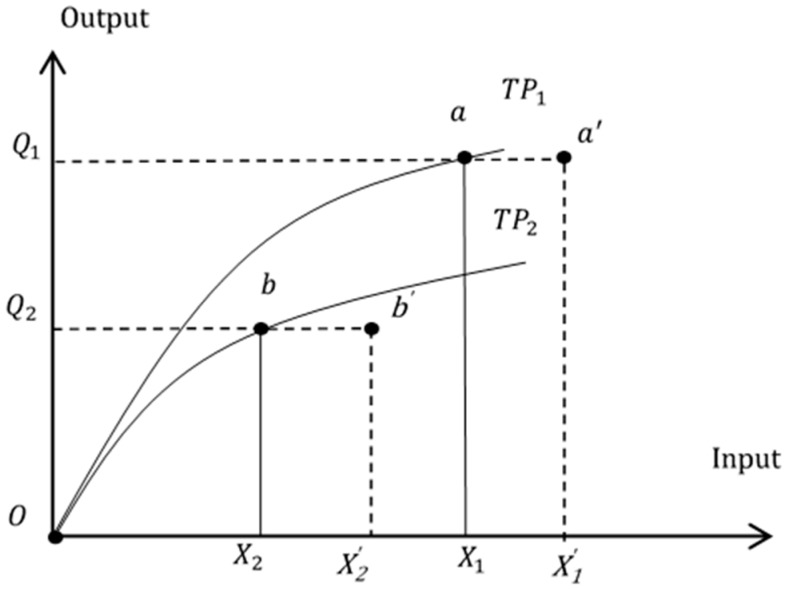
Production frontiers of two senior care facilities, *TP*_1_ and *TP*_2_.

To illustrate the technical efficiency, we draw the production frontiers of the facilities of two different operating scales in the market, *TP*_1_ and *TP*_2_ in Figure 1. Assuming there is no accreditation system or competition, organization 1 produces maximum output *Q*_1_ given input *X*_1_, and its average productivity is calculated as *AP*_1_ = *OQ*_1_*/OX*_1_. Similarly, for organization 2, given output *Q*_2_ and input *X*_2_, the average productivity is *AP*_2_ = *OQ*_2/_*OX*_2_. If government accreditation or competition is considered, to obtain good accreditation results, organizations might increase input resources to serve the same number of inpatients (output) under the inefficiency quality competition assumption. That is, input *X*_1_ increases to *X’*_1_ and *X*_2_ to *X’*_2_ and outputs remain at *Q*_1_ and *Q*_2_. Thus, excessive resource inputs exist: $\Delta X_{1}={\mathrm{OX}}_{1}^{\prime }-{\mathrm{OX}}_{1}$ and $\Delta X_{2}={\mathrm{OX}}_{2}^{\prime }-{\mathrm{OX}}_{2}$ . Technical efficiency equations of the two facilities can be derived as below.

(1a) $$TE_{1}=\frac{OX_{1}}{OX_{1}^{\prime }}<1$$

(1b) $$TE_{2}=\frac{OX_{2}}{OX_{2}^{\prime }}<1$$

Following the example in Figure 1, we construct null hypotheses for the study as shown in Equation (2). Hypotheses H1–H5 examine whether general care facilities and nursing homes differ in terms of quality of care and technical efficiency. Hypothesis H6 tests the existence of inefficient quality competition. If null hypothesis H6 is rejected in the empirical studies, we can conclude that facilities devote excessive resources to better accreditation scores without any improvement in productivity/efficiency. That is, quality competition is inefficient in the industry.

(2) H1: The input levels of general care facilities and nursing homes are the same
H2: The output levels of general care facilities and nursing homes are the same
H3: The productivity of general care facilities and nursing homes are the same
H4: The quality of care of general care facilities and nursing homes are the same
H5: The technical efficiency of general care facilities and nursing homes are the same
H6: Quality of care is positively related to technical efficiency

### 2.2. Data Envelopment Analysis (DEA)

DEA analyses are frequently used in the literature to evaluate the technical efficiency of a facility within a group of subjects. For example, the radical primal DEA models as in Charnes, Cooper, and Rhodes [31] (CCR DEA model) or the non-radical slacks-based measure (SBM) DEA models as in Tone [32]. Each has distinctive characteristics and terms of use. CCR DEA models aim to minimize inputs in order to achieve maximum production efficiency. However, all inputs have the same relative weight. SBM DEA models, on the other hand, are able to adjust inputs to different combinations of weights and consequently, SBM models do not apply to products which have fixed input–output ratios. Tone and Tsutsui [33] introduced an epsilon-based measure (EBM) DEA which utilizes an affinity index between inputs and outputs. Thus, EBM takes into account diversity of input/output data and their relative importance for measuring efficiency. To illustrate the advantage of adopting an EBM DEA model in this study which combines both radical and non-radical of measures, in this section we compare the primal CCR DEA model, the slacks-based measure (SBM) DEA model, and our proposed EBM DEA model under constant returns to scale- and input-oriented assumptions in mathematical formulations and in a graph.

● CCR Model

Based on the CCR model as in Charnes et al. [31], to evaluate the technical efficiency $\left(\theta^{*}\right)$ of a decision-making unit (organization) given input and output (*x*_0_, *y*_0_), we can write the linear programming equations as follows where λ is the intensity vector of weight of inputs and $s^{-}$ is non-radical slacks.

(3) $$\theta^{*}=\underset{0,\lambda ,s^{-}}{min}\;\theta \;subject\;to\;\theta x_{0}=X\lambda -s^{-}\;y_{0}\leq Y\lambda ,\;\lambda \geq 0,\;s^{-}\geq 0$$

● SBM Model

**Figure 2:**
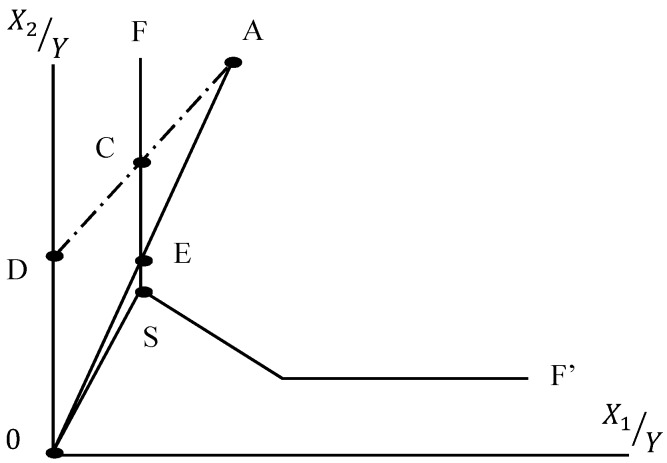
Comparison of Charnes, Cooper, and Rhodes (CCR), slacks-based measure (SBM), and epsilon-based measure (EBM) data envelopment analyses.

Cooper et al. [34] formulated the following slacks-based measure (SBM) DEA model as in Equation (4). $\tau^{*}$ is the technical efficiency of decision making unit (DMU) given input and output. Tone [32] proved that $\;\theta^{*}$ , the technical efficiency of the CCR model in Equation (3), is equal to or greater than $\tau^{*}$ , i.e., $\tau^{*}\leq \theta^{*}$ . We will compare the results in a graph later in Figure 2.

(4) $$\tau^{*}=\min\;\;1-\frac{1}{m}\overset{m}{\underset{i=1}{\sum }}\frac{s_{i}^{-}}{x_{i0}}\;subject\;to\;\;x_{i0}=\overset{n}{\underset{j=1}{\sum }}x_{ij}\lambda_{j}+s_{i}^{-}\;\;\left(i=1,\ldots ,m\right),\;y_{i0}\leq \overset{n}{\underset{j=1}{\sum }}y_{ij}\lambda_{j}\;\;\left(i=1,\ldots ,s\right),\;\lambda_{j}\geq 0\;\left(\forall j\right),\;\;s_{i}^{-}\geq 0\;\left(\forall i\right)$$

● EBM Model

In our empirical studies, we follow the DEA model of Tone and Tsutsui [33] and construct an epsilon-based measure data envelopment analysis which is input oriented and has constant returns to scale assumptions, as shown in Equation (5). Let $w_{i}$ be the weight of input *i*. The advantage of the EBM model relies on the introduction of parameter $\varepsilon_{X}$ , combining radical technical efficiency θ in Equation (3) and non-radical slacks $\overset{m}{\underset{i=1}{\sum }}\frac{W_{i}^{-}X_{i}}{X_{io}}$ in Equation (4) into technical efficiency $\gamma^{*}$ , and an affinity index between inputs and outputs along with principal component analysis. When $\varepsilon_{X}=0$ , the EBM model becomes the CCR model; and when $\varepsilon_{X}=1$ , the formulation becomes the SBM model.

(5) $$\gamma^{*}=\underset{0,\lambda ,s^{-}}{min}\left(1-\varepsilon_{X}\right)\theta +\varepsilon_{X}\overset{m}{\underset{i=1}{\sum }}\frac{W_{i}^{-}X_{i}}{X_{io}}\;subject\;to\;\;x-x\lambda =0,\;\;x=\theta x_{0}-s^{-},\;\;Y\lambda \geq y_{0},\;\;\lambda \geq 0,\;s^{-}\geq 0$$

To compare the above three models, assume a decision-making unit A which requires two types of input resources (*X*_1_, *X*_2_) and produces the single output *Y*. The efficiency frontier is drawn as FF’ in Figure 2. Under the assumptions of the CCR model, DMU A must maintain the same weight of input combination. To improve technical efficiency, A will move along $\vec{AD}$ and decrease inputs to point C to maximize objective function. Thus, the technical efficiency of DMU A under the CCR model is:

(6) $$\theta =\frac{DC}{DA}$$

However, under the assumptions of the SBM model, the weight of the input combination can be changed. DMU A can move from point C to point S to further maximize the objective function, and the technical efficiency of the SBM model becomes:

(7) $$\tau =\frac{OS}{OA}$$

The affinity index of the EBM model enables DMU A to consider the diversity combination of inputs and outputs, and move between point C and point S. Therefore, DMU A can produce at point E and the technical efficiency of the EBM model γ is written as:

(8) $$\gamma =\frac{OE}{OA}$$

From Equations (6)–(8), we observe that

(9) θ≥γ≥τ

### 2.3. Metafrontier Efficiency Analysis (MTE)

Considering facility differences in the scale or nature of operations, we combine the previously introduced EBM DEA and metafrontier efficiency approach to evaluate and compare efficiencies including group technical efficiency, the technical gap ratio, and metafrontier efficiency across groups of facilities. The detailed steps are as follows.

1. Calculate group technical efficiency $(GTE_{i}^{j}$ ) for each facility by means of the EBM DEA model in Equation (5), where $GTE_{i}^{j}$ represents the technical efficiency of facility *i* in group *j*.
2. Obtain the technical gap ratio ( $TGR_{i}^{j}$ ). $TGR_{i}^{j}$ is an estimate of the ratio of the efficiency of facility *i* to the overall efficiency of all facilities. Overall efficiency is obtained by inserting the target input and output values obtained from Step 1 of all groups into Equation (5).
3. Calculate the metafrontier efficiency $MTE_{i}^{j}$ of each facility by combining results from step 1 and 2 by the following equation:
(10) $$MTE_{i}^{j}=GTE_{i}^{j}\times TGR_{i}^{j}\leq 1$$

**Figure 3:**
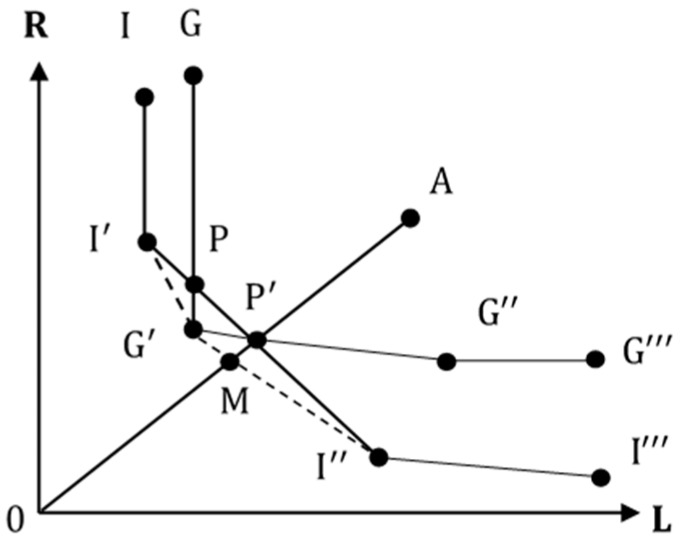
Comparison of pool frontier and metafrontier.

To illustrate the advantage of using metafrontier efficiency analysis in a graph, assume there are two distinct groups of facilities: group I & G. We draw two separate frontiers II’I’’I’’’ and GG’G’’G’’’ as in Figure 3. Suppose point A represents a facility in group I. When the distinct characteristics of each group are considered and metafrontier analysis is applied, the resulting metafrontier is defined as ${\mathrm{II}}^{\prime }G^{\prime }I^{\prime \prime }I^{\prime \prime \prime }$ which is composed of the lower bounds of the two frontiers. We can write the group technical efficiency of A = $OP^{\prime }/OA$ and the technical gap of A as $OM/OP^{\prime }$ *.* By Equation (10), metafrontier efficiency of A is calculated as $MTE=GTE\times TGR=\left(\frac{OP^{\prime }}{OA}\right)\times \left(\frac{OM}{OP^{\prime }}\right)=\frac{OM}{OA}$ . However, if we evaluate the technical efficiency of all facilities without distinction, the pool frontier is ${\mathrm{II}}^{\prime }{\mathrm{PG}}^{\prime }P^{\prime }I^{\prime \prime }I^{\prime \prime \prime }$ . As seen in the figure, the pool frontier shows concavity at point P and $P^{\prime }$ . Efficiency is not monotonic. For A to move to point $P^{\prime }$ , the pool frontier efficiency of A becomes $\frac{OP^{\prime }}{OA}$ instead of $\frac{OM}{OA}$ .

### 2.4. Iterative Seemingly Unrelated Regression

To evaluate the relation between quality of care and metafrontier efficiency, we built a regression model based on the duality theory. We assumed senior care facilities are non-profit in nature, which means that the operating objective of senior care facilities is to minimize costs rather than maximizing profits. Suppose we have three types of input resources and a single output *Q*. Let *P_i_* be the price of input *I_i_*, *i* = 1–3. We can construct the following objective function:

$$Min\;P_{1}I_{1}+P_{2}I_{2}+P_{3}I_{3}\;\;s.t.\;\bar{Q}=f\left(I_{1},I_{2},I_{3}\right)$$

After obtaining the solution of input prices and first order conditions for output $\bar{Q}$ , based on Shephard’s Lemma, we write a demand equation for price and output as

$$X_{i}=X_{i}\left(P_{1},P_{2},P_{3},\bar{Q}\right),\;\;\;\;\;i=1,2,3$$

In the empirical studies, we are not able to acquire cost information. Instead, we utilize quality of care (*CQ*), operating scale (*CAR*), and accommodation rate (*BED*) to reflect price. Therefore, the demand equation becomes the following.

$$X_{i}=X_{i}\left(CQ,CAR,BED,\bar{Q}\right)\;\;\;\;i=1,2,3$$

However, the underlying assumption behind the above equation is that every facility is operating on the frontier and resources are fully utilized. In this study, we aim to test whether operating efficiency differences exist among facilities as shown in Figure 1 and Equation (10). Hence, we propose a metafrontier function (*MTE*) as a function of quality of care, operating scale, accommodation rate, output and demand as follows,

$$MTE=f\;\;(CQ,CAR,BED,\bar{Q},X_{1},X_{2},X_{3})$$

Based on the above equation, to estimate the relation among quality of care, inputs, and metafrontier efficiency, we constructed four regression Equation (11a–d). In the census data, quality of care is evaluated by senior facility accreditation results which are classified as A, B, and C. Thus, let CQH and CQM be the dummy variable to represent quality rating A and B respectively. DSP is the number of direct care personnel, ISP is the number of indirect care personnel, FLO is floor area, APE is actual number of people accommodated, and BED is the accommodation rate. CAR is the operating scale. CAR = 1 represents senior facilities of the general care type and CAR = 0 represents nursing homes. For detailed definitions of variables, please refer to Table 1.

(11a) $$LOG\left(DSP_{i}\right)=\alpha_{1}+\alpha_{2}CQH_{i}+\alpha_{3}\left(CQH_{i}*CAR_{i}\right)+\alpha_{4}CQM_{i}+\alpha_{5}(CQM_{i}*CAR_{i})\;+\alpha_{6}LOG\left(APE_{i}\right)+\alpha_{7}BED_{i}+\varepsilon 1_{i}$$

(11b) $$LOG\left(ISP_{i}\right)=\beta_{1}+\beta_{2}CQH_{i}+\beta_{3}\left(CQH_{i}*CAR_{i}\right)+\beta_{4}CQM_{i}+\beta_{5}(CQM_{i}*CAR_{i})\;+\beta_{6}LOG\left(APE_{i}\right)\;+\;\beta_{7}BED_{i}+\varepsilon 2_{i}$$

(11c) $$LOG\left(FLO_{i}\right)=\gamma_{1}+\gamma_{2}CQH_{i}+\gamma_{3}\left(CQH_{i}*CAR_{i}\right)+\gamma_{4}CQM_{i}+\gamma_{5}(CQM_{i}*CAR_{i})\;+\gamma_{6}LOG\left(APE_{i}\right)+\gamma_{7}BED_{i}+\varepsilon 3_{i}$$

(11d) $$MTE_{i}=\theta_{1}+\theta_{2}CQH_{i}+Q_{3}\left(CQH_{i}*CAR_{i}\right)+Q_{4}COM_{i}+Q_{5}\left(CQM_{i}*CAR_{i}\right)+Q_{6}LOG\left(APE_{i}\right)\;+Q_{7}BED_{i}+Q_{8}LOG\left(DSP_{i}\right)+Q_{9}LOG(ISP_{i})+Q_{10}LOG(FLO_{i})+\varepsilon 4_{i}$$

In the empirical studies, if the error terms in Equation (11a–d) show contemporaneous relations, we propose to utilize an iterative seemingly unrelated regression, ISUR (Zellner [35]) to enhance accuracy in the regression estimations. If no contemporaneous equation exists, we simply adopt the least square method to solve the above regression models. To test contemporaneousness, a Breush–Pagan Lagrange multiplier test (LM) is utilized as in Equation (12). *LM* is a $\chi^{2}$ distribution with degree of freedom = 6 and $\rho_{ij}^{2}$ is the square of correlation coefficient of residuals.

(12) $$LM=T\overset{4}{\underset{i=2}{\sum }}\overset{i=1}{\underset{j=1}{\sum }}\rho_{ij}^{2}\sim \chi^{2}$$

In addition, by applying the chain rule to regression Equation (11a–d), we can further determine whether accreditation ratings (CQH & CQM) have a direct or mediating effect, which is an indirect influence through changes in input resource allocations, on MTE efficiency. Calculations are shown in Equations (13a)–(14f). If the total effects of the direct and mediating effects in the empirical studies show negative numbers suggesting that quality of care and MTE efficiency are negatively related and null hypothesis H6 in Equation (2) is rejected, it can then be concluded that inefficient quality competition exists among the senior care facilities.

For general care facilities: 

Mediating Effect of CQH:

(13a) $$\left(\alpha_{2}+\alpha_{3}\right)\theta_{8}+\left(\beta_{2}+\beta_{3}\right)\theta_{9}+\left(\gamma_{2}+\gamma_{3}\right)\theta_{10}$$

Direct Effect of CQH:

(13b) $$\theta_{2}+\theta_{3}$$

Total Effect of CQH:

(13c) $$\left(\alpha_{2}+\alpha_{3}\right)\theta_{8}+\left(\beta_{2}+\beta_{3}\right)\theta_{9}+\left(\gamma_{2}+\gamma_{3}\right)\theta_{10}+\theta_{2}+\theta_{3}$$

Mediating Effect of CQM:

(13d) $$\left(\alpha_{4}+\alpha_{5}\right)\theta_{8}+\left(\beta_{4}+\beta_{5}\right)\theta_{9}+\left(\gamma_{4}+\gamma_{5}\right)\theta_{10}$$

Direct Effect of CQM:

(13e) $$\theta_{4}+\theta_{5}$$

Total Effect of CQM:

(13f) $$\left(\alpha_{4}+\alpha_{5}\right)\theta_{8}+\left(\beta_{4}+\beta_{5}\right)\theta_{9}+\left(\gamma_{4}+\gamma_{5}\right)\theta_{10}+\theta_{4}+\theta_{5}$$

For nursing homes:

Mediating Effect of CQH:

(14a) $$\alpha_{2}\theta_{8}+\beta_{2}\theta_{9}+\gamma_{2}\theta_{10}$$

Direct Effect of CQH:

(14b) $$\theta_{2}$$

Total Effect of CQH:

(14c) $$\alpha_{2}\theta_{8}+\beta_{2}\theta_{9}+\gamma_{2}\theta_{10}+\theta_{2}$$

Mediating Effect of CQM:

(14d) $$\alpha_{4}\theta_{8}+\beta_{4}\theta_{9}+\gamma_{2}\theta_{10}$$

Direct Effect of CQM:

(14e) $$\theta_{4}$$

Total Effect of CQM:

(14f) $$\alpha_{4}\theta_{8}+\beta_{4}\theta_{9}+\gamma_{2}\theta_{10}+\theta_{4}$$

## 3. Empirical Results

This section presents empirical results utilizing the census data from Taiwan’s senior care industry. Section 3.1 explains variables and the source of the data collected in the study, Section 3.2 examines the null hypotheses, Section 3.3 evaluates efficiencies using DEA models, Section 3.4 shows the results of metafrontier efficiency analysis, and finally Section 3.5 presents the results of the regression models.

### 3.1. Variable and Data Descriptions

Our data was collected from the Senior Citizens Care Facility Accreditation Report issued by the Ministry of the Interior of Executive Yuan, Taiwan. Evaluated subjects included all levels of public and private senior care facilities established before December 2008. The accreditation process was implemented during 2008–2010 and input and output data of all facilities was collected in 2011. Therefore, there existed a one-way causality between accreditation ratings and input and output variables. The accreditation report evaluated and classified senior facilities into five levels: the highest quality level A, B, C, D, and the lowest quality level E. However, only the input and output information of those facilities rated above C, 128 facilities altogether, is public and available in the official census database. After removing 36 subjects with missing data, and excluding one subject that exceeded its lawful accommodation capacity, we have 91 senior care facilities including 52 general care facilities and 39 nursing homes. Table 1 defines input and output variables. Table 2 shows the summary statistics of the variables. Note that the actual accommodation, APE, and all input resources, DSP, ISP, and FLO, of general care facilities, on average, are much greater than those of nursing homes.

### 3.2. Test of Hypotheses

This section presents the test results for the null hypotheses in Equation (2). Table 3 shows *t*-tests for the input and output variables. It can be seen again that the means of three inputs, DSP, ISP, and FLO, and the output, APE, of general care facilities are all greater than those of nursing homes. Therefore, we reject null hypotheses H1 and H2 in Equation (2). General care facilities and nursing homes are different in operating scales. We also calculated productivity, which equals the actual output/input, and found that general care facilities have higher productivity in direct care personnel (DSP) while nursing homes have higher productivity in indirect care personnel (ISP) and floor area (FLO). These two types of facilities have distinct productivities. Thus, null hypothesis H3 is rejected.

Table 4 summarizes the official accreditation results of 91 senior care facilities. General care facilities in general have higher rankings than nursing homes. 92.3% of general care facilities are ranked above B. However, only 76.9% of nursing homes are ranked above B. We conducted the $\chi^{2}$ test of Mann–Whitney ( $\chi^{2}=$ 2.057) and found that the quality of care of these two types of facilities are different. Thus, null hypothesis H4 is rejected. To sum up this section, it can be observed that general care facilities and nursing homes are different in input resources, output, productivity, and quality of care. One must separately evaluate these two types of facilities when evaluating operating efficiency. Hence, in the next sections, we conduct and present efficiency results via data envelopment analysis and metafrontier efficiency analysis.

### 3.3. Data Envelopment Analysis

Before conducting data envelopment analysis, a necessary step is to ensure the input and output variables of our data increase monotonically, and the correlation of the coefficient must be positive. Table 5 shows that DSP, ISP, and FLO are all positively related to the output variable APE. We can then proceed to conduct DEA models. To show differences in employing different DEA models, we compare the results of the CCR, SBM, and EBM DEA models in Table 6. It is observed that the average and medium technical efficiency values of the EBM DEA model in three panels are in between those of the CCR and SBM models, which confirms our illustrations in Figure 1 and Equation (9). Since the CCR and SBM models are special cases of the EBM DEA model, in the next section we will evaluate group technical efficiency, technical gap ratio, and metafrontier efficiency based upon the EBM model.

### 3.4. Metafrontier Efficiency Analysis

**Figure 4:**
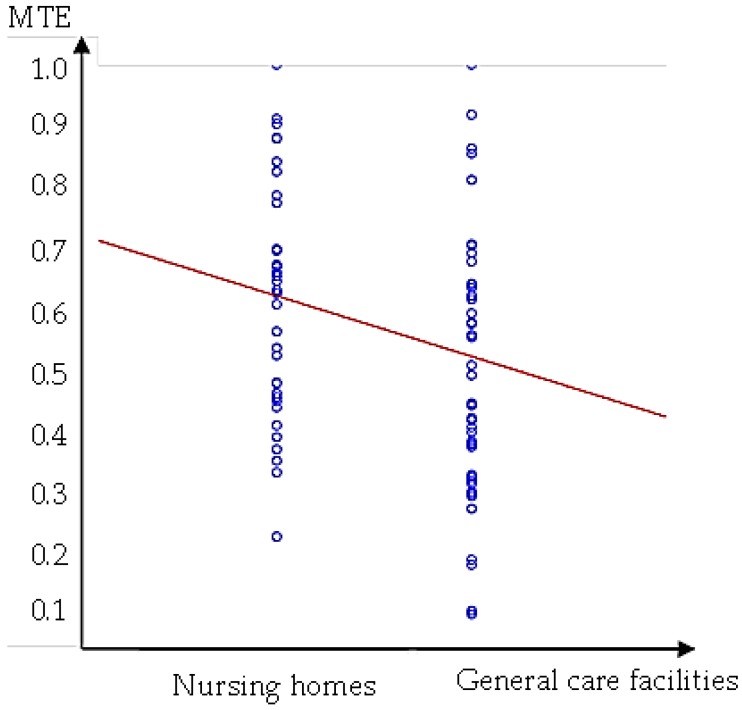
Metafrontier efficiency (MTE) results of different types of senior facilities.

In this section, we first compare results in terms of group technical efficiency (GTE) and pool technical efficiency (PTE), then we calculate the technical gap ratio and metafrontier efficiency, and finally, conduct statistical analysis. The results in Table 7 shows that GTE does not equal PTE. Efficiency differences exist among senior facilities. The use of PTE analysis alone cannot characterize individual differences. Therefore, we apply the metafrontier analysis to separately consider general care facilities and nursing homes. The results are shown in Table 8. The medium GTE of all samples is 0.65, which means that there is still room for improvement, amounting to 35% to achieve complete efficiency in senior care facilities. Nursing homes have better efficiency than general care facilities in terms of GTE. The average technical gap ratio (TGR) of the two types of facilities (0.898 & 0.997) is close to 1, indicating that both types of facilities are very close to the efficiency frontier and nursing homes are closer to the efficiency frontier than general care facilities. The average MTE of general care facilities is smaller than that of nursing homes. Figure 4 illustrates MTE values for both types of facilities. The negative regression line suggests that general care facilities on average are less efficient in the industry. Meanwhile, it is observed that there is a huge gap between the minimum and maximum of the GTE, TGR, and MTE values. In other words, those inefficient facilities have to make tremendous efforts to close the gap with benchmark facilities in the industry. To conclude our summary of the results in Table 8, it is found that nursing homes on average have higher efficiencies than general care facilities and that the efficiency disparity among general care facilities is greater than that of nursing homes. Table 9 lists the percentage of facilities that achieved the benchmark score/full efficiency (i.e., GTE, TGR, or MTE = 1). Note that about 95% of nursing homes reach TGR = 1, which means that 95% of nursing homes operate on the efficiency frontier. However, only 8% of nursing homes achieve GTE = 1. The combined effect of the two efficiencies, MTE, showed that only 5% of nursing homes operate on the frontier. GTE indicates the internal management efficiency of a facility within the same type of facility, while TGR evaluates both types of facilities, which means that the impact of external environmental factors in the industry is included. Thus, we concluded that the overall inefficiency of nursing homes was a result of internal management rather than external environmental factors. We also notice that of all benchmark facilities, only one is publicly operated. The rest are privately owned facilities. This might be because of additional profit-driven incentives that private facilities have for maintaining operating efficiency.

Table 10 shows the results of the $\chi^{2}$ test of Mann–Whitney utilizing the medium of three efficiencies (GTE, TGR, and MTE). The medium group technical efficiency (GTE) of general care facilities is smaller than that of nursing homes; however, the $\chi^{2}$ test is not significant. The TGR of both types of facilities are significantly close to full efficiency. The MTE efficiency of nursing homes is significantly greater than that of general care facilities. The results in Table 10 verify that the two types of senior facilities have distinct efficiency frontiers. Therefore, the null hypothesis H5 in Equation (2) is rejected.

### 3.5. Regression Results

Table 11 shows the results of the least square regression model in Equation (11a–d) which examine the relation among quality of care, inputs, and metafrontier efficiency. We conducted several tests to assess model fit. Variance inflation (VIF) of the four regression equations is less than five except for VIF4 of the Logarithm of APE, LOG (APE) = 5.077. Small VIF values indicate that multicollinearity does not exist among variables. The results of the regression specification error test (RESET) suggested that some other variables might exist that needed further investigation when conducting the MTE regression equation, Equation (11d). In testing goodness-of-fit, the Breush–Pagan Lagrange multiplier test, LM value, showed no contemporaneous relationships among residuals; the least square method was then applied to run regression models. System R^2^ = 0.984, suggesting that the model fits the data well.

**Figure 5:**
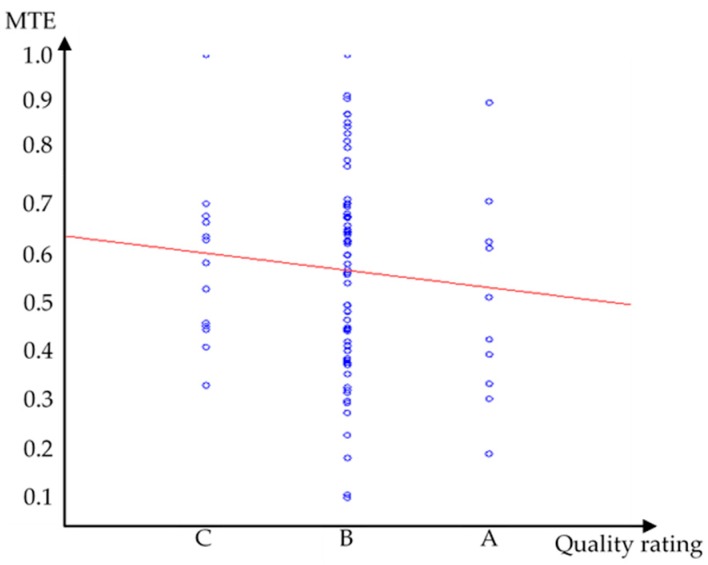
The relation between MTE results and quality rating.

We observe that quality rating is positively related to input resources. For general care institutions, quality rated A (COH) and B (CQM) are both positively related to floor area (FLO). For nursing homes, A rated nursing homes are positively related to direct care personnel (DSP). However, for B rated nursing homes, quality rating does not show significant impact on input resources. It is also observed that the actual number of people accommodated (APE) is positively related to all three input resources (DSP, ISP and FLO). Increasing APE encourages institutions to devote more resources into operations. However, increasing input resources (DSP, ISP and FLO) has a negative impact on efficiency as shown in the metafrontier efficiency (MTE) column. Meanwhile, the quality rating is negatively related to MTE for general care facilities rated A and B, but positively related to nursing homes rated A. Figure 5 shows the regression line between MTE and the quality rating of all facilities, and a negative relationship between efficiency and quality rating can be clearly observed.

To further clarify whether the quality rating has a direct effect on efficiency or it first influences input resource allocation and then, in turn, affects efficiency, we applied chain rules to regression equations to define the direct, mediating and total effects of quality rating on MTE. The results in Table 12 show that, for A- and B-rated (CQH and CQM) general care facilities, the direct effects of quality rating on efficiency are not significant. However, the rating has shown a negative mediating impact on efficiency. Combining the mediating and direct effects, the total effects for general care facilities are still negative, which means that quality rating influences input resource allocation in a way that is not beneficial to improving efficiency. For nursing homes rated B, however, the influences are not significant. For nursing homes rated A, the mediating effect is negative and the direct effect is positive and the total effect ends up being insignificant. Therefore, from Table 11 and Table 12, we conclude that inefficient quality competition only exists in general care facilities. Thus, we can only partially reject null hypothesis H6.

## 4. Conclusions

This study combines two data envelopment analysis (DEA) methods: Epsilon-based measure (EBM) and metafrontier efficiency analysis (MTE) to estimate the operating efficiency of senior care facilities in Taiwan followed by a regression model to investigate the relationship between quality of care and operating efficiency. Data in the empirical studies includes census data from 91 senior facilities, 52 general senior care facilities and 39 nursing homes, obtained from the Senior Citizens Care Facility Accreditation Report issued by the Executive Yuan of Taiwan. The results are summarized below.

1. The results of the EBM data envelopment analysis, which allows flexibility to adjust input and output ratios to fit various types of operations, proved to be different from the results of conventional data envelopment analysis. We showed that general care facilities and nursing homes have distinct efficiency frontiers. Therefore, it is necessary to conduct metafrontier efficiency analysis, which allows us to evaluate and compare the operating efficiency across different groups of senior facilities.
2. The metafrontier efficiency results showed that there is, on average, room for improvement amounting to 45% for general care facilities and 36% for nursing homes to achieve complete metafrontier efficiency. The operating efficiency of general care facilities, with greater operating scales and better accreditation ratings on average, is inferior to that of nursing homes in Taiwan.
3. The results of the regression model indicated that a tradeoff relationship exists between quality of care and metafrontier efficiency among general care facilities. General care facilities are observed to devote more input resources in order to obtain better ratings in the accreditation reports/quality of care; however, metafrontier efficiency is not improved but rather reduced. The inefficiency quality competition hypothesis is sustained for general care facilities. This observation corresponds to the inferior operating efficiency of general care facilities as noted in observation two. However, the same inference cannot be drawn for nursing homes. The small number of observations of nursing homes in the census data might be the reason that the relation between quality and efficiency in the empirical studies was not significant. The relatively small number of nursing homes in Taiwan might be due to two factors in our observations: the relatively small population of the country and the heavy reliance on home/resident care due to cultural concept of the youth bearing the responsibility for caring for the elderly in the family in Taiwan. Especially, the latter factor explains why nursing homes, which care for people who still have self-care ability, are not popular in the senior care system.
4. The metafrontier efficiency results also showed that of all benchmark facilities (efficiency value = 1), of the eight facilities with a technical gap ratio = 1 and four facilities with metafrontier efficiency = 1, only one facility is publicly operated. This might be because privately-owned facilities have more financial incentives to achieve operating efficiency than public organizations.

Taiwan’s government has paid special attention to the senior care industry in recent years. However, our studies have showed that there is still a large gap to improve in terms of quality of care and operating efficiency. This study was restricted to public census data. We propose to collect panel data with financial information and extend the time period of data coverage for future research so as to better observe the long-term impact of government policies and changes in quality in the industry. Furthermore, active aging is also a focus for senior citizens. Technology developments that help to improve healthy lifestyles such as information and communication technology, healthcare assistance, mobile medical devices, and so forth in senior care facilities also have a profound influence in the industry. We recommend that Taiwan’s government include related indices in senior care facility accreditation to better reflect health care quality in the industry.

## Figures and Tables

**Table 1:** Variable definitions.

Variable	Notation	Unit	Definition
Input variables			
Direct nursing personnel	DSP	People	Nursing personnel, social workers, caregivers and other professionals related to the services provided
Indirect personnel	ISP	People	Administrative personnel include administrators, technicians, pharmacists, sanitary personnel, etc.
Floor area	FLO	Square meters	Floor area of the buildings
Output variables			
Actual accommodation	APE	People	Actual accommodation at the time of accreditation report

**Table 2:** Summary statistics.

Variables	Statistics	General Care Facilities	Nursing Homes	Total
DSP	Average	42.596	28.949	36.747
	Maximum	117.000	114.000	117.000
	Minimum	8.000	7.000	7.000
	Standard deviation	29.417	19.586	26.429
ISP	Average	13.385	7.385	10.813
	Maximum	67.000	26.000	67.000
	Minimum	2.000	1.000	1.000
	Standard deviation	11.264	5.413	9.653
FLO	Average	2.228	0.487	1.482
	Maximum	10.500	4.580	10.500
	Minimum	0.109	0.023	0.023
	Standard deviation	2.327	0.833	2.028
APE	Average	152.712	78.333	120.835
	Maximum	481.000	179.000	544.000
	Minimum	10.000	17.000	20.000
	Standard deviation	116.589	45.969	99.824

**Table 3:** Tests of the significance of differences.

	General Care Facilities	Nursing Homes	*t*-Test
Panel A: Means			
DSP	42.596	28.949	(2.508 **)
ISP	13.385	7.385	(3.068 ***)
	General care facilities	Nursing homes	*t*-Test
FLO	2.228	0.487	(4.459 ***)
APE	152.712	78.333	(3.766 ***)
Panel B: Productivity			
DSP/APE	3.783	2.875	(2.851 ***)
ISP/APE	13.921	14.428	(−0.205 ***)
FLO/APE	141.481	392.589	(−5.352 ***)

** and *** represents 5% and 1% level of significance.

**Table 4:** Summary of accreditation results.

Ranking	A	B	C
General care facilities	11.54%	80.77%	7.69%
Nursing homes	10.26%	66.67%	23.08%
Total	10.99%	74.73%	14.29%

**Table 5:** Correlation of coefficient.

**General Care Facilities**			
APE	DSP	ISP	FLO
	0.838	0.608	0.387
	(10.853 ***)	(5.422 ***)	(2.970 ***)
**Nursing Homes**			
APE	DSP	ISP	FLO
	0.659	0.362	0.436
	(5.326 ***)	(2.360 **)	(2.943 ***)

** and *** represent 10%, 5% and 1% levels of significance respectively.

**Table 6:** Comparisons of CCR, SBM, and EBM DEA models.

	CCR	SBM	EBM
General care facilities			
Average	0.636	0.559	0.621
*F*-test	(1.431)		
Medium	0.656	0.546	0.641
$\chi^{2}$	(2.923)		
Nursing homes			
Average	0.684	0.502	0.644
*F*-test	(8.860 ***)		
Medium	0.706	0.435	0.665
$\chi^{2}$	(13.608 ***)		
Total			
Average	0.656	0.535	0.631
*F*-test	(7.303 ***)		
Medium	0.682	0.492	0.650
$\chi^{2}$	(11.810 ***)		

*** represents 1% level of significance.

**Table 7:** Group technical efficiency (GTE) and pool technical efficiency (PTE).

	GTE	PTE
Average	0.631	0.559
t test	(2.313 **)	
Medium	0.650	0.534
$\chi^{2}$	(2.659 **)	

** represents 5% level of significance.

**Table 8:** Results of the metafrontier efficiency analysis.

	GTE	TGR	MTE
General care facilities			
Average	0.621	0.898	0.548
Medium	0.641	0.925	0.527
Maximum	1.000	1.000	1.000
Minimum	0.159	0.659	0.150
Nursing homes			
Average	0.645	0.997	0.642
Medium	0.665	1.000	0.665
Maximum	1.000	1.000	1.000
Minimum	0.270	0.886	0.270
Total			
Average	0.631	0.941	0.589
Medium	0.650	1.000	0.598
Maximum	0.941	1.000	1.000
Minimum	0.159	0.659	0.150

**Table 9:** Percentage of benchmark facilities in each sample.

	GTE = 1	TGR = 1	MTE = 1
General care facilities	12%	23%	6%
Nursing homes	8%	95%	5%

**Table 10:** Mann–Whitney $\chi^{2}$ test of technical efficiencies.

Technical Efficiency	General Care Facilities	Nursing Homes	$\chi^{2}$
GTE	0.641	0.665	(0.599)
TGR	0.925	1.000	(5.971 ***)
MTE	0.527	0.665	(2.346 ***)

*** represents 1% level of significance.

**Table 11:** Results of the least square regression model.

	Equation	Log(DSP)	VIF1	Log(ISP)	VIF2	Log(FLO)	VIF3	MTE	VIF4
Variable									
CQH general care facilities		0.023 (0.093)	2.479	0.315 (0.806)	2.479	1.222 ** (2.408)	2.479	−0.109 ** (−2.011)	2.639
CQM general care facilities		−0.031 (−0.330)	1.565	0.115 (0.744)	1.565	0.698 *** (3.477)	1.565	−0.061 *** (−2.768)	1.774
CQH nursing homes		0.348 * (1.664)	2.958	0.298 (0.879)	2.958	0.334 (0.761)	2.958	0.084 * (1.810)	3.076
CQM nursing homes		−0.029 (−0.229)	2.038	0.289 (1.431)	2.038	0.297 (1.131)	2.038	0.039 (1.423)	2.103
BED		0.002 (0.768)	1.109	−0.003 (−0.839)	1.109	−0.033 *** (−7.721)	1.109	−0.0006 (−0.975)	1.849
LOG (APE)		0.685 *** (12.392)	1.320	0.582 *** (6.500)	1.320	1.096 *** (9.422)	1.320	0.478 *** (20.153)	5.077
LOG (DSP)								−0.306 *** (−12.992)	3.803
					
LOG (ISP)								−0.097 *** (−6.485)	2.053
					
LOG (FLO)								−0.082 *** (−7.272)	3.850
					
CONSTANT		0.166 (0.637)		−0.649 (−1.537)		−3.567 *** (−6.509)		−0.329 *** (−4.774)	
RESET *F*-test (1,80)		0.462		4.079		0.004		20.141 ***	
R^2^		0.722		0.456		0.721		0.847	
Goodness of fit		LM = 9.314				System R^2^ = 0.984			

Note: () indicates *T*-test. *, **, and ***denotes 10%, 5%, and 1% level of significance.

**Table 12:** Impact of quality of care on MTE efficiency.

	Mediating Effect	Direct Effect	Total Effect
CQH (general care facilities)	−0.301 *** (14.688)	−0.025 (0.268)	−0.325 ** (13.859)
CQM (general care facilities)	−0.103 ** (4.453)	−0.022 (0.568)	−0.125 ** (5.254)
CQH (nursing homes)	−0.163 ** (4.042)	0.084 * (3.276)	−0.078 (0.718)
CQM (nursing homes)	−0.044 (0.819)	0.039 (2.026)	−0.004 (0.006)

Note: () represents $\chi^{2}$ values, and *, **, and ***denotes 10%, 5%, and 1% level of significance.
